# Collectanea Medica

**Published:** 1814-03

**Authors:** 


### COLLECTANEA MEDICA,

CONSISTING OF

### ANECDOTES, FACTS, EXTRACTS, ILLUSTRATIONS, QUERIES, SUGGESTIONS, &c.

RELATING TO THE

### *History or the Art of Medicine, and the Auxiliary Sciences.*

WE are induced to transcribe the following interesting particulars respecting the state of medicine in Spain, about twenty years since, in the hope that some of our correspondents, who have lately visited the Peninsula, will favour us with an account of the improvements which have taken place in the condition and the practice of the physicians of that country, since the time when Mr. Townsend travelled.

"The science and practice of medicine are at the lowest ebb in Spain, but more especially in the Asturias. *Fiat venesectio* is still the favourite prescription, notwithstanding the ridicule of Le Sage, and the serious reasoning of Feyjoo. When the fond husband meets the physician in the street, and urges him to step in to see his wife, *Sangrado* pulls out at

 once once his list of patients and his watch, tells him that he cannot stop one moment, orders him instantly to fetch the surgeon, and to have her blooded, promising faithfully to see her in the space of half an hour. Palsies most undoubtedly are frequent, but it is by no means clear, that these are always caused by plethora, although in many cases they certainly originate from fulness. *Sangrado*, however, has such a dread of palsy, that he bleeds his patient into a dropsy, or leaves him to languish between life and death, a prey to the most gloomy of all diseases to which humanity is subject."

"Of diseases, the most endemical are intermittent and putrid fevers. These arise from the proximity of an extensive swamp, containing many hundred acres, which might easily be drained, so as to produce the most luxuriant crops. In the year 1785, during the three autumnal months, they lost two thousand five hundred persons, and the succeeding year two thousand three hundred more; yet the Almojar remains undrained. Government, indeed, exerted its authority, but not in the most effectual manner, for the relief of the inhabitants.

"When the report of this calamity had reached the court, an order was dispatched to the physicians, that no other medicine should be administered to the sick, than the famous one prescribed by Don Joseph Masdeval, and called by him his opiate, of which the following is the formula:

℞ Sal absinth,
—Ammoniac optime depurati āā ʒ i.
Tartari Stibiati, termino clariori Tartari Emetici gr. xviij. triturentur per horæ quadrantem, deinde adde & optime misceantur Pulv. Cort. Peruv. ℥i.
Syr. absinth q. s. fiat Opiata.

"Of this he gives one-sixth part every two hours, with one spoonful of the following mixture:

℞ Aq. viper ℥v.
Aq. benedict Rulandi termino clariori Vini Emetici ℥j.
Cremor Tartari pulv. ʒj. m.

"With these medicines he interposes plenty of broth, and continues to use them till the patient is restored to health.

"In a conversation I had with him at court, he informed me, that the common operation of these medicines was at first to act as an emetic or cathartic, often bringing away lumbrici; but, being continued, they relieved the stricture on the external surface of the body, promoted perspiration, and acted sometimes as a diuretic. He assured me, that in the most desperate cases, the disease had given way at the end end of four days after he had begun to administer his medicines; and he did me the honour to shew me a variety of attestations from medical men in almost every part of Spain.

"That I might have no doubt of the true nature of the disease, he related the usual symptoms, such as, in the beginning, a remarkable prostration of strength, with intense pain both of the head and of the back; intolerable thirst; the tongue foul, dry, black, chopped, and trembling, when protruded; pulse small, hard, quick, and intermitting; parotid glands swelled; urine limpid at first, but turbid in the progress of the disease; respiration difficult; the white of the eyes become red; petechial spots on the arms and breast; hands trembling; watchfulness at first, followed by propensity to sleep perpetually without consciousness of having slept; delirium; noise in the ears, followed by deafness; involuntary tears; coldness of the extremities; quivering of the under lip; and, if the patient were ill treated, death.

"From this description, there could be no doubt of the disease; but, as to the operation of the medicines, that certainly will admit of some discussion. On the common principles of chemistry it is evident, that a double decomposition takes place, and that the tartar emetic is reduced to an inert calx. I must acknowledge, that when first I was informed of this curious medicine, I was inclined to think, that the tonic power of the bark enabled the stomach to bear this extraordinary quantity of tartar emetic, but on more mature consideration it seems clear, that, being decomposed, this active medicine has lost its efficacy; and I am confirmed in this idea by a fact related to me by Dr. Masdeval, when I had the honour to meet him at the Escurial. He had prescribed this opiate to a monk, who was in the last stage of a *typhus* or putrid fever; but the nurse, by mistake, gave the whole quantity at once, thus administering eighteen grains of tartar emetic at one dose, yet without any other visible effect than abating the violence of all his symptoms. I am therefore satisfied, that the cleansing of the alimentary canal must be attributed to the emetic wine, and that the operation of the famous opiate would be nearly the same either with or without the stibiated tartar, and must be ascribed wholly to the bark.

"The physicians of Carthagena were willing to allow this medicine all the credit which was due to it, and to prescribe no other whenever they should be convinced that this might be used with safety; but to be precluded in all cases from the use of other remedies, they thought, would be unreasonable.

unreasonable. They therefore sent their remonstrances to court; but in answer, there came an express order from the king, that they should be subject to the intendant of the dock-yard, and should prescribe according to his directions.

"On the receipt of this mandate from the court, the intendant immediately assembled the physicians, and made known the royal pleasure, informing them, that in case of disobedience, the prisons were prepared, and the guards in waiting to execute his orders. They expostulated, but to little purpose; and being told that nothing short of absolute submission would be accepted, they consented to prescribe the opiate in all cases, and, to evince their sincerity, they signed a certificate, that no other medicine was so efficacious as this recommended by the king.

"The people, however, were not so submissive to the royal mandate, and knowing that the physicians were engaged not to vary their prescriptions according to the exigency of the case, and the variety of diseases by which they might be attacked, they absolutely refused to send for medical assistance, and resolved to take their chance for life or death. When, therefore, information was carried back to court, that the physicians were likely to be starved, and the people to die for want of their advice; the minister relented, and agreed to compromise the matter, leaving the sons of Æsculapius at liberty to follow their own judgment for the citizens at large, and compelling them to administer no other medicine, beside the opiate, to all the patients in the royal hospital.

"This perhaps is the first instance of despotic power controlling the functions of physicians, and prescribing uniformity to that class of citizens in the line of their profession."

"I have observed in general, that the physicians, with whom I have had occasion to converse, are disciples of their favourite doctor Piquer, who denied, or at least doubted of, the circulation of the blood. Yet they begin to get acquainted with the names of Van Swieten, Hoffmann, Sauvage, Gaubius, de Haen, and Cullen. They have indeed laboured under the greatest disadvantages in their education, and in the want of encouragement when they entered upon practice, receiving little money, and less honour, in the way of their profession. In their medical classes they had no dissections, no experiments in chemistry, and for botany they were unacquainted with Linnæus. These defects will now be remedied. But even in the present day, the fee of a physician is, two pence from the tradesman, ten ten pence from the man of fashion, and nothing from the poor. Some of the noble families agree with a physician by the year, paying him annually four score reals, that is, sixteen shillings, for his attendance on them and on their families.

"They all acknowlege that the monks are more liberal than people of the first fashion, more especially if confidence and secrecy are needful.

"In point of honour, no class of citizens meets with less respect than the physicians; but in proportion as the nation shall acquire wealth, they will rise up in consequence, and be regarded with esteem.

"Of one thing, which in Spain is required from chirurgeons and physicians, I have never been able to find any one who could give me a satisfactory account. Before they enter into their profession, they are obliged to swear, that they will defend the immaculate conception of the Blessed Virgin. This requisition is the more extraordinary, because that point is not universally agreed upon, even between Catholics themselves; yet many centuries may pass before the medical tribe will be freed from this unreasonable imposition. To give due weight to the sanction of an oath, every country should purge away those which are become obsolete, but more especially such as are universally regarded as absurd."

"Of threescore physicians settled at Barcelona, Don Francisco Sanponts and Don Francisco Salva are the most distinguished, and have the most extensive practice. One of them favoured me with a sight of his list. He had visited more than forty patients in the morning, and he was to see as many before he went to bed. Among these were many merchants, manufacturers, and officers; yet he did not expect to receive a hundred reals, that is twenty shillings, for the whole practice of the day.

"Although not rich, they had occasion, a few years since, to shew a high and independent spirit, for which they deserve the highest commendation. When General O'Neille was governor, (A. D. 1784,) the putrid fever raged in Catalonia, as in Arragon and other provinces of Spain. The physicians, summoned by the governor, like those of Carthagena, were required to engage, that from thenceforth they would prescribe no medicine beside the famous opiate recommended by Dr. Masdeval. Not satisfied with this, the governor had prepared a certificate, similar to the one produced at Carthagena, for them to sign. The doctors Salva and Sanponts, in the name of all the rest, remonstrated; but could obtain no other answer, than that the king would have it so, and that the prison doors stood open to receive them. Our chieftains, however, not to be intimidated, continuing firm to their resolution, and being well supported by their corps, at last came off triumphant, and were permitted to prescribe whatever medicines they thought proper. The general, although as a soldier he had been accustomed to obedience, yet being gentle and discreet, he chose rather to report the matter to the court, than at once to carry his threats into execution. Here the matter rested.

"Dr. Masdeval, in his publication, claims the invention of this opiate, and represents it not merely as a specific in putrid fevers, but as a panacea, infallible in all kinds of fever, and a sovereign remedy in every disease incident to the human frame. But as the physicians of Barcelona were by no means satisfied of this, they resisted his pretensions; and as some of them had noticed this famous opiate in the *Journal de Medecine*, so far back as A.D. 1769, they denied him the merit of invention. In reality, this formula was known and described under the appellation of Boucher's opiate, and the nature of the decomposition taking place on the admixture of the various articles was well described in the Journal of 1778.

"In conversing with these physicians, I was struck with the number of lunatics under confinement in the several provinces of Spain; and when I returned to England, I compared their account of Catalonia with the government returns. By these it appears, that in Arragon the number is two hundred and forty-four; in Catalonia, one hundred and fourteen; in Valencia, one hundred and twenty-one; in Andalusia, ninety-nine; in Granada, forty-one; in Toledo, forty-two; in the province of Leon, two; and in Avila, one. In the other interior provinces no mention is made of any. Thus stands the fact; but as for the foundation of this difference between the maritime and the inland provinces in this respect, neither they nor any one with whom I have conversed on the subject, could suggest any thing worthy of remark. I have, therefore, been contented simply to state the fact, and leave it as I found it."—*Townsend's Journey through Spain, London,* 1791.

CRITICAL

